# Hemolysis at low blood flow rates: in-vitro and in-silico evaluation of a centrifugal blood pump

**DOI:** 10.1186/s12967-020-02599-z

**Published:** 2021-01-05

**Authors:** Malte Schöps, Sascha H. Groß-Hardt, Thomas Schmitz-Rode, Ulrich Steinseifer, Daniel Brodie, Johanna C. Clauser, Christian Karagiannidis

**Affiliations:** 1 Department of Cardiovascular Engineering, Institute of Applied Medical Engineering, Medical Faculty, RWTH Aachen University, Pauwelstrasse 20, 52074 Aachen, Germany; 2 Center for Acute Respiratory Failure, Columbia University College of Physicians and Surgeons/NewYork-Presbyterian Hospital, New York, NY USA; 3 Department of Pneumology and Critical Care Medicine, Cologne-Merheim Hospital, ARDS and ECMO Centre, Kliniken der Stadt Köln GmbH, Witten/Herdecke University Hospital, Ostmerheimer Strasse 200, 51109 Cologne, Germany

**Keywords:** Hemolysis, Off-design point, Pediatric patients, ECMO, ECCO_2_R, Centrifugal blood pumps, Extracorporeal circulation

## Abstract

**Background:**

Treating severe forms of the acute respiratory distress syndrome and cardiac failure, extracorporeal membrane oxygenation (ECMO) has become an established therapeutic option. Neonatal or pediatric patients receiving ECMO, and patients undergoing extracorporeal CO_2_ removal (ECCO_2_R) represent low-flow applications of the technology, requiring lower blood flow than conventional ECMO. Centrifugal blood pumps as a core element of modern ECMO therapy present favorable operating characteristics in the high blood flow range (4 L/min–8 L/min). However, during low-flow applications in the range of 0.5 L/min–2 L/min, adverse events such as increased hemolysis, platelet activation and bleeding complications are reported frequently.

**Methods:**

In this study, the hemolysis of the centrifugal pump DP3 is evaluated both in vitro and in silico, comparing the low-flow operation at 1 L/min to the high-flow operation at 4 L/min.

**Results:**

Increased hemolysis occurs at low-flow, both in vitro and in silico. The in-vitro experiments present a sixfold higher relative increased hemolysis at low-flow. Compared to high-flow operation, a more than 3.5-fold increase in blood recirculation within the pump head can be observed in the low-flow range in silico.

**Conclusions:**

This study highlights the underappreciated hemolysis in centrifugal pumps within the low-flow range, i.e. during pediatric ECMO or ECCO_2_R treatment. The in-vitro results of hemolysis and the in-silico computational fluid dynamic simulations of flow paths within the pumps raise awareness about blood damage that occurs when using centrifugal pumps at low-flow operating points. These findings underline the urgent need for a specific pump optimized for low-flow treatment. Due to the inherent problems of available centrifugal pumps in the low-flow range, clinicians should use the current centrifugal pumps with caution, alternatively other pumping principles such as positive displacement pumps may be discussed in the future.
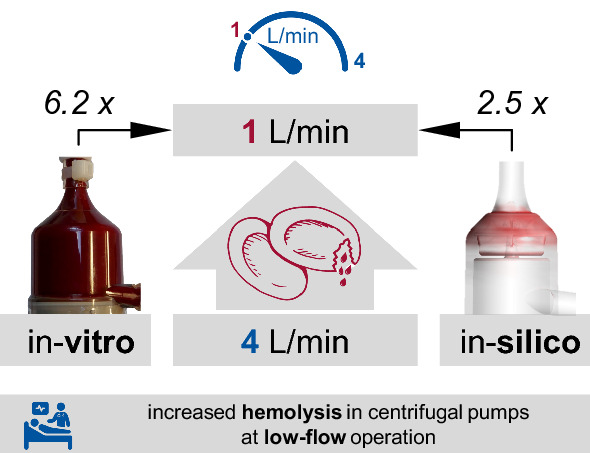

## Introduction

The use of extracorporeal membrane oxygenation (ECMO) in adult patients has been increasing rapidly in recent years [1]. ECMO therapy has become an established alternative as a rescue therapy in the treatment of severe forms of the acute respiratory distress syndrome (ARDS) with promising case series of extracorporeal CO_2_ removal (ECCO_2_R) in chronic obstructive pulmonary disease (COPD) [2–4]. ECMO use in neonatal or pediatric patients, or the use for ECCO_2_R, were considered the key low-flow applications in this study [5]. The use of ECMO in children with a weight below 10 kg increased on average by 2.2 % per year between 2011 and 2016. The predominant use of roller pumps switched to centrifugal pumps during this time period [6].

Centrifugal blood pumps as central elements of ECMO therapy provide good results in the high-flow operating ranges of 4 L/min–8 L/min of blood flow. However, during low-flow applications, i.e. neonatal or pediatric ECMO or ECCO_2_R, the operating ranges are markedly lower at 0.5 L/min–2 L/min. These low-flow operating points contribute to adverse events such as increased hemolysis, platelet activation and bleeding complications [7, 8]. Furthermore, this may be a particularly important issue in vulnerable pediatric patients, where blood trauma should be carefully avoided [6, 9–14].

Centrifugal blood pumps were originally designed for a specific operating point, but in clinical practice, they are used within a wider operating range. Increasing pump speed and blood flow rate is associated with an increase in blood damage. However, it is misleading to extrapolate that a lower pump flow leads to less blood damage [10, 15]. To date, there are only a few pumps that have been designed for these requirements or tested in the low-flow range [16–19]. This highlights the urgent need to focus on technical implications associated with the use of current centrifugal blood pumps in different low-flow ranges.

We hypothesize that the hemolytic potential of centrifugal blood pumps at a low-flow applications (1 L/min) is higher in contrast to high-flow operation (4 L/min), which present with lower hemolysis. In the current work, we evaluated the hemolysis of a blood pump at both operating points in-vitro and compared the results with in-silico computational fluid dynamics (CFD) simulations and flow paths within the pump.

## Materials and methods

The in-vitro test setup was performed according to [20], and the numerical in-silico methods for the CFD investigations used the DP3 (Xenios AG, Heilbronn, Germany) as an example of a blood pump in current clinical use.

### Flow loop design

**Fig. 1:**
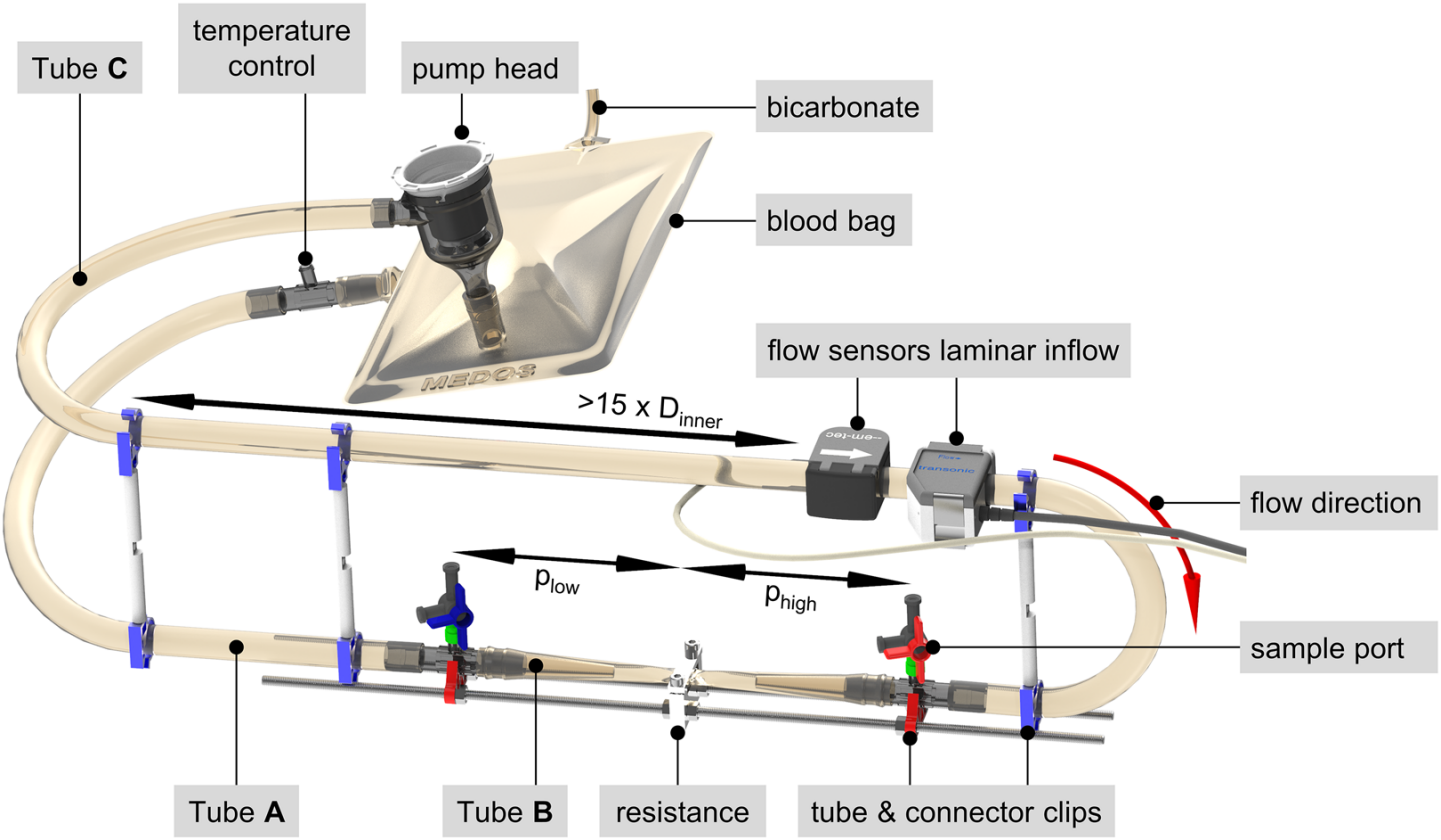
In-vitro test setup of the flow loop according to [20]. Red indicates the high pressure measuring point, blue indicates the low pressure measuring point. p_low_ and p_high_ highlight the equal distances between the pressure measuring points and the resistance. For the flow sensors a laminar inlet distance is essential, which should be at least 15 times the inner diameter of the tube, which was 33 times during in-vitro tests of this study

To perform in-vitro hemolysis testing, three identical flow loops plus one reference reservoir—for static conditions—were prepared for each test day. The test loops were assembled by three segments of PVC tubing (3/8″ × 3/32″ RAUMEDIC-ECC-noDOP^®^, Raumedic AG, Germany). A smaller welded medos soft blood bag (Medos Medizintechnik AG, Germany) with 204 mL ± 28 mL was used as a closed reservoir and connectors with and without Luer (FLEIMA-PLASTIC GmbH, Germany) were used in between. Spacers with 3D printed tube and connector clips were added to keep the bending radii of the tubes equal and to guarantee the laminar flow inlet distance for the flow sensors in each simultaneously running flow loop, see Fig. 1. The tests were repeated four times at 4 L/min (n = 4), and five times at 1 L/min (n = 5).

To avoid results that would correlate with the specific pump head, the serial numbers were tracked and the pump heads rotated through the three flow loops at each repetition.

### Blood collection and, anticoagulation

Five one-liter blood bottles were prepared with 1.8 mL glucose (0.5 mg/mL), 1.6 mL gentamycin (10 mg/mL), and 50 ml NaCl (0.009 g/mL). While 15,000 IU/L heparin was initially used at both low and high-flow, heparin was reduced to 4500 IU/L due to the high activated clotting time (ACT) values. Each bottle was filled with one liter of porcine blood at the slaughterhouse and immediately transferred to the blood lab, filtered through a nylon stocking and pooled in a five-liter bag (Nutrimix^®^, B. Braun Melsungen AG, Germany). The hematocrit of the pooled blood was determined after gentle, but thorough mixing.

### Flow loop preparation

The flow loops were prefilled with saline solution (< 500 mL) to displace all remaining air. As much saline solution as possible was drained from the flow loops and replaced with pooled blood. Based on the weighted remaining volume of saline solution, the necessary blood volume to achieve a total volume of 454.1 mL in the first in-vitro test and in all following ones 483.94 mL ± 1.25 mL (mean ± SD) at a hematocrit of 31.16 % ± 1.12 % was added to the flow loops. Subsequently, all air was removed from the flow loops. The pumps were operated at low speeds of 1252 rpm on average, achieving a homogeneous mixing of the blood. Subsequently, a sample was drawn in order to verify the hematocrit, determine the standard base excess and finally adjust the standard base excess to 0 mmol/L ± 5 mmol/L using 8.4 % sodium bicarbonate solution (Fresenius Kabi, Germany). A reference reservoir was filled with the same pooled blood, therefore having equal hematocrit, and placed in one heated water bath under static conditions. With the exception of the pump head and the flow sensor, each flow loop was placed in a water bath (Lauda, Germany) at 37 °C (36.78 °C ± 0.57 °C). As soon as the base excess was adjusted, the pump speed was set to the target speed and pressure difference was adjusted with the Hoffmann hose clamp as a resistance. Samples were taken from the flow loops every 60 min. Sampling volume was 4 mL with a previous discard of 2 mL. After six hours test duration, the pump heads were thoroughly cleaned with a pepsin citrate solution, rinsed with de-mineralized water, dried and stored for the next test.

Multi-center studies reveal widely varying results due to many patient-specific parameters. In order to avoid this, we focused on high repeatability in the in-vitro tests. Further details on sample and data acquisition can be found in the Appendix.

### Pump operating points

In this study, an upper pressure head target of 250 mmHg was chosen to be consistent with typical CO_2_ removal applications [10]. Two different pump flows were chosen, as those typically used in low-flow and high-flow applications of ECMO or ECCO_2_R [21, 22].

The low-flow operating point was set to 1 L/min (0.96 L/min ± 0.04 L/min, mean ± SD) and the pressure head to 250 mmHg (249.77 mmHg ± 5.58 mmHg), requiring a pump speed of 6250 rpm (6259 rpm ± 26 rpm). When the operating point was set to 4 L/min (3.94 L/min ± 0.04 L/min) using the same rotational speed of 6250 rpm (6226 rpm ± 78 rpm), the pressure drop measured 206.61 mmHg ± 7.32 mmHg.

In order to achieve high comparability between in-vitro and in-silico, the CFD simulations were adjusted according to the in-vitro average values of hemoglobin and hematocrit.

### Hemolysis measurement

In order to compare the pumps operating points based on a clinically relevant parameter, we evaluated the hemolysis rate, i.e. how much delta plasma free hemoglobin (ΔpfHb) in mg per min was produced in the flow loops. In the comparison of pumps, both the Normalized Index of Hemolysis (NIH) and the Modified Index of Hemolysis (MIH) are established. In this study, we refer mainly to MIH, as it is normalized to the total hemoglobin content. This is meaningful since the latter varies from in vitro test to in vitro test. The formulas are based on the ASTM F1841-97(2017) standard [20] and on the publication of Adachi et al. [23] described in the Appendix, compare equations (1), (3) and (4).

1 $$MIH = \frac{{\Delta fHb \cdot V \cdot \left( {\frac{100 - Ht}{100}} \right)}}{Q \cdot T \cdot Hb}$$

Compare [20]

$\Delta fHb$ increment of plasma free hemoglobin concentration in ${\text{mg}}\;{\text{dL}}^{ - 1}$

$V$ whole blood volume in flow loop in ${\text{mL}}$

$$Ht$$ hematocrit in $$\%$$

$Q$ flow rate in ${\text{L}}/{\text{min}}$

$T$ sampling period in ${ \text{min} }$

$Hb$ total hemoglobin in ${\text{g}}/{\text{dL}}$

The free plasma hemoglobin concentration was evaluated photometrically at 540 nm and 680 nm as reference wavelengths according to the gold standard, DIN 58931:2010-08, using the cyanmethemoglobin (HiCN) method [24, 25]. For further information on double determination and other aspects of this procedure, the reader is referred to the Appendix.

### Numerical CFD setup and in-silico hemolysis evaluation

The simulation setup was aligned with Gross-Hardt et al. [10] to ensure comparability of the results. Micro-CT scans were performed to determine the geometry of the DP3 pump head. The geometry mesh was generated using tetrahedral elements, which merge into 18 refining prismatic wall layers with a prism growth rate of 1.2, resulting in 6.73 million mesh-elements for the 18.1 mL internal blood volume of the pump head [26].

In order to determine hemolysis numerically, the following simulation parameters are required: The time during which blood is exposed to a scalar shear stress, called exposure time. The three-dimensional shear stress tensor is converted into a scalar shear stress and, using the present flow, transformed into a hemolysis index via the well-known power-law relationship. The numerical hemolysis prediction was analogously performed to Gross-Hardt et al. [10]. By means of the commercial element-based finite volume method (ebFVM), the solver CFX (ANSYS CFX, ANSYS, Inc., Canonsburg, PA, USA) and the sliding mesh approach, the transient Reynolds-averaged Navier–Stokes (RANS) momentum and mass equations was iteratively solved. Using scalar variable residuals and stabilized predictions of the simulation parameters, the convergence was monitored until the simulation parameters showed stable results. In order to achieve transient stability, at least five complete impeller rotations were computed before transient result were averaged over the following two rotations. In all simulations, the time step was chosen proportional to 5° of the impeller rotation, resulting in 504 iterations for each operation point, of which 144 were used for statistical result averaging. Blood density was adjusted to 1059 kg/m^3^, while the viscosity was modelled as shear-dependent [27]. Blood-specific and varying parameters, like hemoglobin and hematocrit were averaged over all in-vitro tests and fed back into the simulation to create consistent conditions.

### CFD—recirculation

Common centrifugal blood pumps have secondary flow within the pump housing to wash out bearings and avoid regions of stagnation, which are prone to thrombus formation. However, the amount of secondary flow is influenced by the geometry and the operating conditions. Recirculation within the pump head is defined as the ratio between the sum of secondary flow in the pump casing, normalized to the flow leaving the pump’s outlet, compare equation (2).

2 $$Recirculation_{ratio} = \frac{{\sum \dot{V}_{secondary} }}{{\dot{V}_{{pump^{\prime}outlet}} }}$$

**Fig. 2:**
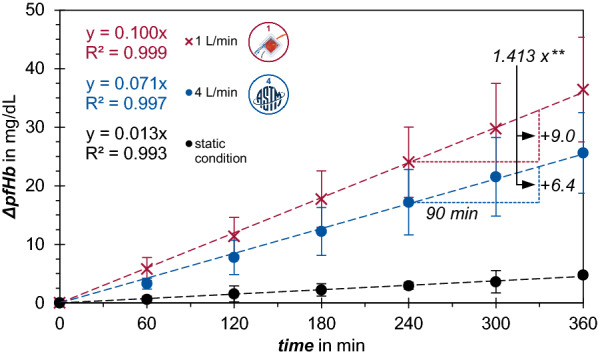
Delta plasma free Hemoglobin (ΔpfHb) concentration over time. The high-flow operating point at 4 L/min is depicted in blue and the low-flow operating point at 1 L/min is depicted in red. The black curve represents the hemolysis taking place without pump under static conditions in an equivalent setup

The slopes in Fig. 2 of the ∆pfHb regression lines were tested for significance with a two sided heteroscedastic *t-*test. Statistical comparison of in-vitro MIH values with in silico MIH values per flow rate were calculated by means of one sample *t*-test (^†‡^). Statistical comparison of mean MIH in-vitro values were calculated by a two-sided heteroscedastic *t*-test (*) (MS Excel, Microsoft). Significance level of p = 0.001 was assumed.

## Results

### Experimental verification—hemolysis

The comparison of hemolysis by means of plasma free hemoglobin (pfHb) at the two operating points from the in-vitro experiments is shown in Fig. 2. The high-flow operating point at 4 L/min is depicted in blue and the low-flow operating point at 1 L/min is depicted in red. The black curve represents the hemolysis taking place without a pump under static conditions in an equivalent setup. The hemolysis is steady over time and shows a slight increase in pfHb, while both operating points show a severe increase in pfHb over time. The increase at 1 L/min is faster and steeper compared to the increase at 4 L/min. The increase of pfHb is represented by a linear curve fit with a coefficient of determination of R^2^ > 0.99 for each operating point.

The slope of the linear curve is a measure for the increase in hemolysis per time unit. With 0.07 mg/(dL min) for the low-flow operating point, the slope is 1.413 fold higher than for the normal operating point with 0.10 mg/(dL min) (p** < 0.001). Hemolysis under static conditions occurs with a gradient of no more than 0.01 mg/(dL min) released pfHB per time unit. The CV (Coefficient of variation) averaged over the duration of 360 min is 32.5 % for the 4 L/min and 27.6 % for the 1 L/min regression line.

**Fig. 3:**
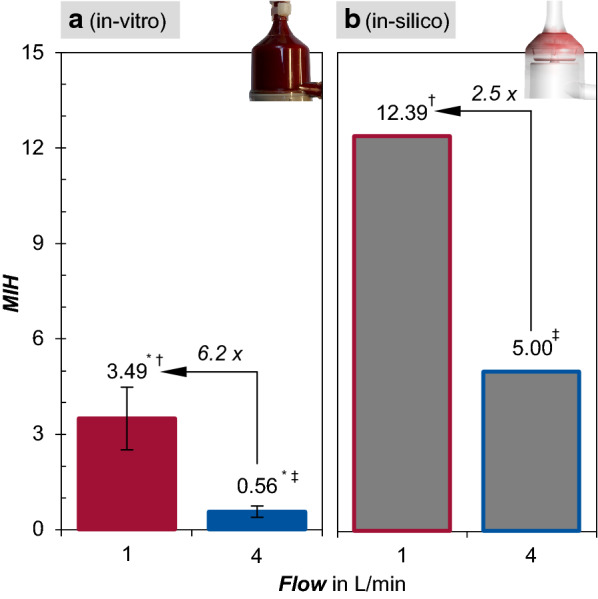
In comparison the in-vitro results represented by Modified Index of Hemolysis (MIH) (**a**) for both 4 L/min and 1 L/min to the corresponding numerically calculated in-silico MIH for both pump operation points (**b**)

Furthermore, the in-silico results differ from the experimental results. Figure 3 compares the in-vitro results in MIH (Fig. 3a) for both 4 L/min and 1 L/min to the corresponding numerically calculated in-silico results (Fig. 3b). The absolute values of both differ significantly (p^†‡^ < 0.001), while showing similar trends. In contrast to the significantly higher relative increase (p* < 0.001) in the in-vitro results, the absolute values are higher in-silico.

While in-vitro values for MIH increase from 0.56 ± 0.18 at 4 L/min to 3.49 ± 0.99 at 1 L/min (mean ± SD), the in-silico MIH is predicted to be significantly higher (p < 0.001) at 5.0 for 4 L/min and 12.39 for 1 L/min. By contrast, the relative increase in-vitro is 6.2 fold higher compared to 2.5 fold for in-silico, compare Fig. 3a, b.

Further data, such as the NIH can be found in the Appendix.

### CFD results—recirculation and associated hemolysis

**Fig. 4:**
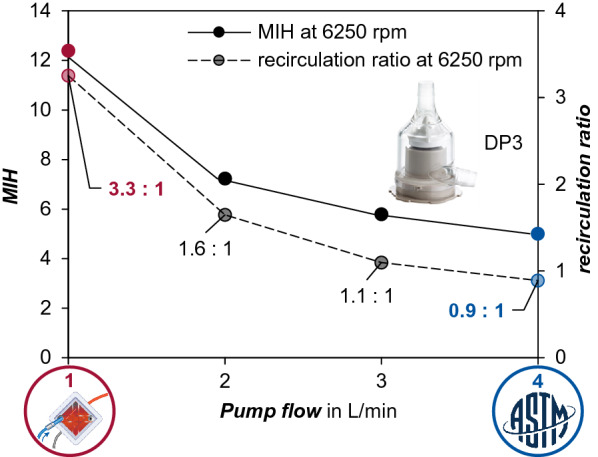
In-silico methods were used to estimate the basic trend of Modified Index of Hemolysis (MIH) and recirculation depending on pump flow. Recirculation CFD results for 2 L/min and 3 L/min extracted from Gross-Hardt et al. [10] (Reproduced with permission from Springer Nature). Each curve represents constant impeller speed to achieve the desired pump pressure head of 250 mmHg at 1 L/min. The recirculation ratio is depicted on the second y-axis above the flow

Figure 4 depicts, on the first y-axis, the MIH determined in-silico, and on the second y-axis, the recirculation ratio over the flow of 1 L/min up to 4 L/min. The blue highlighted operating point represents a high-flow operating point for ECMO and corresponds to the operating point during manufacturer testing for approval. The test procedure is reduced to one operating point, but the pump, when used clinically, does so across an operating range of flows. The operating point highlighted in red is within the typical low-flow range. The steepest increase is obvious in the lower-flow below 2 L/min, both in recirculation and in hemolysis, expressed in MIH.

The trend of the recirculation curve closely follows the increase in MIH towards lower flows. While at 4 L/min the recirculation ratio is 0.9:1, meaning that 3.6 L recirculate inside the pump head while 4 L leave the pump at the outlet. In contrast, at 1 L/min the recirculation ratio is 3.3:1, meaning 3.3 L recirculate inside the pump head, while only 1 L leaves the pumps outlet.

**Fig. 5:**
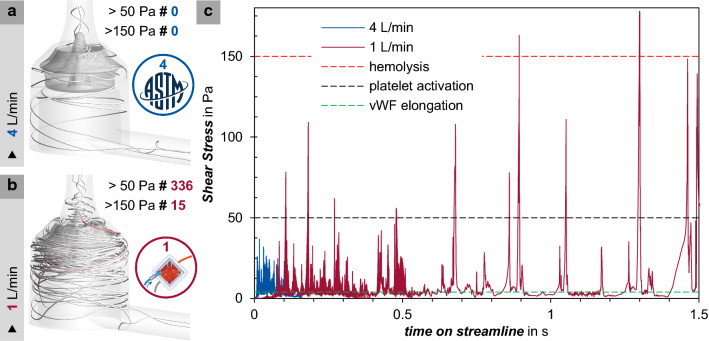
Exemplary streamline with number of threshold exceedances, at 4 L/min (**a**) at 1 L/min (**b**) averaged shear stress of a particle over 1000 streamlines at 4 L/min and 1 L/min displayed over time, with abbreviation: von Willebrand factor (vWf) (**c**)

In Fig. 5a the streamline depicts the trajectory of a tracked particle, such as a red blood cell, at a flow rate of 4 L/min. The trajectory of a particle at 1 L/min is shown in Fig. 5b. The shear stress average over 1000 streamlines is plotted in Fig. 5c against the time a particle follows the streamline.

The increased recirculation (Fig. 5b), leads to multiple exposure of the single blood components. The tracked particle experiences different shear stress over time in the pump head. Comparing a particle at 4 L/min and 1 L/min, respectively, the particle remains in the pump head 9.3 times longer at the low-flow rate. Additionally, the threshold values for von Willebrand factor (vWf)-elongation, platelet activation and hemolysis are exceeded more often at 1 L/min flow. When crossing the threshold value between 5 Pa and 10 Pa, the vWf is already been unfolded. While the limit for platelet activation (> 50 Pa) is not exceeded once at 4 L/min, on average, it is already exceeded 336 times at 1 L/min [28–32]. Similar results are observed for the hemolysis threshold value (> 150 Pa) [32, 33]. This threshold is again not even exceeded once at 4 L/min, but exceeded 15 times at 1 L/min.

Looking at the residence time of a particle within the pump head, the difference between both flow rates is quite noticeable. At a flow rate of 1 L/min this already exceeds 1.51 s, whereas at 4 L/min it is only 0.16 s.

## Discussion

In this study, the increase in hemolysis is evaluated both in vitro and in silico using the DP3 centrifugal pump at two operating points. The in-vitro tests performed in this study demonstrate a significantly higher increase (p* < 0.001) in hemolysis from 4 L/min to 1 L/min with the same centrifugal pump and significantly lower (p^†‡^ < 0.001) absolute values in hemolysis than CFD. Hemolysis was measured via the released plasma-free hemoglobin and expressed by both ΔpfHb and MIH. Although anticoagulation differed slightly during in-vitro test, results are in line with the remaining tests. Therefore, we assume, that small variations in the blood conditions have no major impact on hemolysis.

While CFD simulations predict that the increase in hemolysis shown as MIH at 1 L/min is 2.5 times higher than at 4 L/min, the in-vitro values significantly increase by a factor of 6.2. Even though both approaches show similar trends, they further reveal the need for improvements and validation of numerical prediction models, especially at the low-flow range.

CFD become continuously more important for the prediction of blood damage. It is generally assumed that CFD simulations tend to overestimate the absolute values of hemolysis, but the qualitative trends between different operating points agree with the in-vitro tests [34]. However, the relative increase of hemolysis from high-flow operating to low-flow operating point is, for example, severely underestimated in CFD by a factor of 3 compared to in-vitro testing in this study.

Therefore, the use at low-flow rates would may have serious consequences for the patient. A key advantage of the numerical simulations is the possibility of investigating flow phenomena within the pump. We observed that the ratio between secondary flows and the actual pumping outflow increases at low-flow rates. While at 4 L/min recirculation is 0.9:1, at 1 L/min the ratio is 3.7 times higher. Even if the absolute values of the in-silico study overestimate the actual hemolysis, they allow for the identification of the increased recirculation during operation at low-flow rates as a plausible explanation for increased hemolysis. Prognosis by CFD is complex and in this study underestimated the relative increase in blood damage [9]. An influence of the slightly higher resistance on hemolysis cannot be avoided in the low-flow experiments at the same speed and could strengthen the trend for increased hemolysis compared to high-flow. While in silico only the pump is simulated with the same boundary conditions as in vitro, the influence of other components in the circuit, for example the resistance, is not modeled and therefore not considered. Furthermore, there are still no robust and established models to predict platelet activation by CFD, and it remains unknown to what extent blood components, e.g. platelets, react during high recirculation and cause additional blood damage in centrifugal blood pumps.

Since the quantity of secondary flow during recirculation could not be determined experimentally until now, it would be essential for a pump comparison to allow for the determination of spatially resolved hemolysis in vitro. In future studies, an approach using ghost cells as a hemolysis indicator in combination with a particle image velocimetry system for flow measurement and detection of spatially resolved hemolysis hotspots could provide more detailed information [35].

Centrifugal blood pumps demonstrate inherent limitations with regard to flow recirculation at low-flow operation. If a hydraulic output is required that demands both low-flow against a high pressure gradient, a centrifugal blood pump will need a certain speed. If the pump flow is below the design point of the pump, high recirculation inside the pump will occur. This phenomenon poses a major problem for pumps in low-flow operation, with regard to blood damage. This study raises awareness of the inappropriate use of centrifugal pumps in low-flow therapy treatment, as long as they are not designed for this specific flow rate. The in-vitro test results indicate the risk of using centrifugal pumps in the low-flow range, even if the pump is approved for a much larger operating range according to the manufacturer’s manual. For this reason, centrifugal pumps in the low-flow range, not operating at their dedicated operating point should be used with caution. However, centrifugal pumps being optimized for the low-flow blood range are urgently needed. Due to the current lack of low-flow centrifugal pumps, displacement pumps may be discussed. An equivalent evaluation in the low-flow area is still pending and existing studies lack comparability due to versatile operating conditions [13].

For now and in future studies, it will be important to better understand the degree to which an increase in blood damage is a serious problem for extracorporeal therapy. In order to assess whether a pump is suitable for treating pediatric or neonatal patients or for low-flow applications, such as ECCO_2_R, it appears reasonable to consider absolute values of blood damage, such as ΔpfHb, and to standardize hemolysis with respect to the time interval of treatment. It is crucial to investigate in detail how much absolute release of pfHb/time a patient can cope with. Additionally, it appears inevitable to review the system limitations of centrifugal blood pumps in general and more systematically. A comprehensive testing of currently available systems needs to be performed, particularly focusing on lower flow rates. In this context, a consensus on a suitable damage index should be established in order to ensure an adequate pump comparison at different operating points for different patient groups with regard to the clinical implications.

In combination with the previous studies of Gross-Hardt et al. [10] and Granegger et al. [9], it appears that current centrifugal blood pumps, such as the DP3 (Xenios AG, Heilbronn, Germany), Rotaflow (Maquet GmbH Getinge Group, Rastatt, Germany), Revolution (LivaNova PLC, London, United Kingdom) or HVAD (Medtronic, Minneapolis, USA), are not recommended for operation at flow rates below 2 L/min. In the manufacturer’s manual, pumps are often assigned a much larger operating range compared to the operating points which are mandatory to test during the approval process. The validity of the operating ranges specified in the manuals is therefore not entirely guaranteed and should be questioned. Therefore, special pumps for these low-flow operating points should be urgently designed and made available on the market.

The study has several limitations. First, the higher resistance, which is a further factor in the increased hemolysis in the low-flow in-vitro experiments, but necessary to keep the rotations constant–as in silico–at both operating points for comparability and validation. Second, during CFX simulation only the pump is computed for five revolutions and the values are averaged over two revolutions. Third, the hemolysis cumulated over time is calculated via the power-law relationship in a hemolysis index. All other components of the circulation such as tubes bending radii, reservoir, resistance and pressure outlets are not considered in silico.

In this study, hemolysis was considered due to the rather straightforward in-vitro evaluation, but blood damage obviously includes other factors besides hemolysis. For example, a high value (> 50 mg/dL) of pfHb causes vWF-mediated platelet adhesion and is considered an independent predictor of mortality with ECMO therapy [36, 37]. The consequences of additional blood damage remain mostly unclear until now. Therefore, monitoring of platelet function and bleeding complications, in addition to hemolysis, should be considered in future studies and should become part of the clinical routine.

## Conclusion

In this study, we compared the hemolysis of a centrifugal pump at two operating points: a high-flow rate of 4 L/min and in a low-flow application at 1 L/min. Both the in-silico and in-vitro results revealed an increase in hemolysis during low-flow operation compared to the high-flow operating point. In contrast, CFD predicted higher absolute hemolysis levels but a smaller increase from low to higher flow, whereas the increase in hemolysis was severely higher in the in-vitro experiments.

This study highlights the underappreciated occurrence of hemolysis when using centrifugal pumps within the low-flow range, as is done in neonatal or pediatric ECMO or with ECCO_2_R. These findings underline the need for the development of pumps optimized for low-flow treatment. However, we recommend using this type of pump only under close monitoring. Due to the inherent problems of available centrifugal pumps for the low-flow range, there is an urgent need to design blood pumps for low-flow applications or to discuss other pumping principles such as displacement pumps, which might be more favorable. The clinical relevance of these findings should be verified in subsequent studies, taking other blood-specific parameters, such as platelet activation or vWf-elongation, into account.

## Figures and Tables

**Fig. 6:**
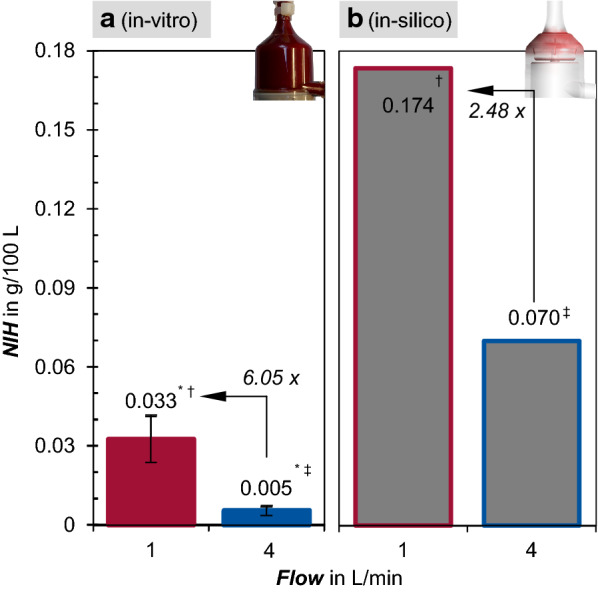
Normalized Index of Hemolysis (NIH) in-silico calculated by CFD (**a**) in-vitro measured NIH (**b**)

## Data Availability

All data generated or analysed during this study are included in this published article and its supplementary information files. The original datasets used and analysed during the current study are available from the corresponding author on reasonable request.

## Appendix

### Flow loop design

The tube segments are assembled as follows: Sections A 500 mm, section B 210 mm and section C 980 mm in length. In addition, four connectors per flow loop are added, each with a length of 77.5 mm, resulting in a total length of 2000 mm of 3/8 inch tube, according to [20], compare Fig. 2.

### Sample and data acquisition

The 4 mL sample was divided between the sampling syringe, a reaction vessel and three citrate flasks, centrifuged at 1500×*g* (Mikro 220, Hettich GmbH & Co. KG, Tuttlingen, Germany). The supernatant of the three citrate flasks was transferred to a new reaction vessel, centrifuged again and finally divided into sample A and B. The samples were subsequently frozen for later photometric analysis.

The blood count was determined via a Sysmex XT-2000iV (Sysmex Deutschland GmbH, Norderstedt, Germany). Blood gas values were determined using an ABL825 FLEX (Radiometer GmbH, Krefeld, Deutschland) and ACT measurements were performed by a Hemochron Jr. Signature Plus (Keller Medical GmbH, Bad Soden, Germany) for each sample. Whereas at the reference reservoir the ACT measurement was performed only at the beginning and after six hours. The pressure was recorded simultaneously via Codan Xtrans^®^ sensors as well as the Codan DPT system (CODAN Medizinische Geräte GmbH & Co KG, Lensahn, Germany), which were calibrated and verified before and after the test using an aneroid sphygmomanometer boso model K (BOSCH + SOHN GmbH u. Co. KG, Jungingen, Germany). The temperature was recorded via a 6-channel handheld data logger RDXL6SD (OMEGA Engineering, Deckenpfronn, Germany), using a Luer-bayonet temperature sensor (Medos, Xenios AG, Heilbronn, Germany). The deviation of the temperature sensor was determined and documented before and after the test using a DKD certificated thermometer (Amarell GmbH & Co. KG, Kreuzwertheim, Germany). As soon as the volume flow no longer matched the operating point, it was readjusted via the resistance and also recorded hourly at each sampling via the ME9PXL16 flow sensors (Transonic Europe B.V., Elsloo, Netherlands) in combination with the T402 Multi-Channel Research Consoles (Transonic Europe B.V., Elsloo, Netherlands) in the Low Flow ¼ Scale setup. In addition, the flow was recorded simultaneously via the 3/8” x 3/32” Clamp-On Transducer (em-tec GmbH, Finning, Germany) in combination with the flow measurement board DigiFlow mini (em-tec GmbH, Finning, Germany).

### Hemolysis evaluation

The below listed formulas base on the ASTM F1841-97(2017) [20] standard and on the publication of Adachi et al. [23]:

3 $$NIH \left[ {{\text{g}}/100\;{\text{L}}} \right] = \frac{{\Delta fHb \cdot V \cdot \left( {\frac{100 - Ht}{100}} \right) \cdot 10^{ - 3} }}{Q \cdot T}$$

compare [20]

4 $${HR \left[ {{\text{mg}}/{ \text{min} }} \right] = \frac{{\Delta fHb \cdot V \cdot \left( {\frac{100 - Ht}{100}} \right) \cdot 10^{ - 2} }}{T}}$$

compare [23]

$HR$ hemolysis rate in ${\text{mg}} \;{ \text{min} }^{ - 1}$

$\Delta fHb$ increment of plasma free hemoglobin concentration in ${\text{mg }}\;{\text{dL}}^{ - 1}$

$V$ whole blood volume in flow loop in ${\text{mL}}$

$$Ht$$ hematocrit in $$\%$$

$Q$ flow rate in ${\text{L}}/{ \text{min} }$

$T$ sampling period in ${ \text{min} }$

$Hb$ total hemoglobin in ${\text{g}}/{\text{dL}}$

The plasma samples were defrosted in a water bath at 37 °C for 8 min. The dilution of the plasma sample to HiCN conversion solution (Hämoglobin (Hb), bioanalytic GmbH, Umkirch/Freiburg, Germany) was in a ratio of 1:5 (v/v) in two microcuvettes (BRAND GmbH + CO KG, Wertheim, Germany) each for double determination [38]. After incubation, the converted free plasma hemoglobin (${\Delta }fHb$) light absorbance was detected by a photometer (Ultrospec 2100, GE Healthcare UK Limited, Little Chalfont, UK). The results were accepted with a coefficient of variation (CV) < 2.87 %, otherwise a second sample was thawed and the determination was repeated, maintaining a CV < 4.98 %.

## Results

### Experimental verification—hemolysis

Figure 6 a depicts hemolysis equivalent to Fig. 3, but in this case using the Normalized Index of Hemolyis (NIH), based on the same in-vitro tests.

## Abbreviations

ECMO: Extracorporeal membrane oxygenation
ECCO_2_R: Extracorporeal carbon dioxide removal
CFD: Computational fluid dynamics
rpm: Revolutions per minute
ACT: Activated clotting time
HI: Hemolysis index
NIH: Normalized index of hemolysis
MIH: Modified index of hemolysis
CV: Coefficient of variation
vWf: von Willebrand factor
